# Relationship between performance, air ventilation efficiency and muscle oxygenation in Firefighters

**DOI:** 10.1186/2046-7648-4-S1-A147

**Published:** 2015-09-14

**Authors:** Philippe Gendron, Eduardo Freiberger, Louis Laurencelle, François Trudeau, Claude Lajoie

**Affiliations:** 1 Laboratoire de physiologie de l'exercice, Département des sciences de l'activité physique, Université du Québec à Trois-Rivières, Trois-Rivières, Canada

## Introduction

Firefighting is a hazardous task associated with a heavy workload where task duration may be limited by air cylinder capacity. Increased fitness may lead to better air ventilation efficiency and task duration at a given heavy work intensity.

## Methods

Thirteen male firefighters (age: 28.4 ± 5.1 years; height: 175.5 ± 4.5 cm; mass: 84.4 ± 9.0 kg; VO_2_peak: 47.8 ± 5.1 mLO_2_.min^-1^.kg^-1^) completed the following tests on 3 different days while wearing firefighting protective clothing (FPC), self-contained breathing apparatus (SCBA) and air cylinder: 1- The graded walking test (GWT) for measuring different physiological parameters while connected to a metabolic system (gas exchanges); 2- The 10 METS treadmill test (T10) designed to measure the time to ventilate air from the cylinder at 10 METS, the intensity needed to complete the fire fit test work simulation described by Deakin et al. (1) within 8 min (2); 3- The simulated work circuit (SWC) to measure the time needed to perform a test mimicking different firefighting tasks while wearing FPC and breathing with the SCBA. Participants performed the SWC as quickly as possible while respecting regulations of the test protocol. Moreover, skeletal muscle oxygenation (deoxyhemoglobin, HHb) was measured during all three tests.

## Results

Firefighters who performed the SWC in a shorter time had lower air cylinder ventilation values on the T10 (*r *= -0.495, *P *< 0.05), better peak oxygen consumption (*r *= -0.924, *P *< 0.001) during the GWT and performed longer until exhaustion on the GWT (*r *= -0.789, *P *< 0.001). Participants who completed the SWC more rapidly and reached a higher VO_2_peak also had lower V_E _and V_E_/VO_2 _values during submaximal workload on the GWT. Moreover, they had greater skeletal muscle deoxygenation during the SWC (HHb, *r *= -0.593, *P *< 0.05).

## Discussion

Greater aerobic fitness was associated with greater air ventilation efficiency of faster firefighters on the SWC. According to Holmér and Gavhed (3), cardiovascular strain is lower in individuals with higher maximal aerobic capacity for a given submaximal intensity. Moreover, correlation between SWC completion time and HHb suggests that better aerobic fitness enhances deoxygenation in the *vastus lateralis *muscle during exercise where the aerobic process of energy production is solicited (4).

## Conclusion

These results demonstrate that the fastest participants on the SWC had better air ventilation efficiency that could prolong interventions in difficult situations requiring air cylinder use. Moreover, the fastest participants had a greater skeletal muscle deoxygenation during the SWC.
